# Effects of Spermidine on Mouse Gut Morphology, Metabolites, and Microbial Diversity

**DOI:** 10.3390/nu15030744

**Published:** 2023-02-01

**Authors:** Dong-Mei Jiang, Ze-Long Wang, Jia-Di Yang, Xin Wang, Chun-Yang Niu, Cheng-Weng Ji, Wei-Kang Ling, Xiao-Guang An, Yong-Ni Guo, Qian Sun, Lin Bai, De-Bing Li, Xiao-Hui Si, Bo Kang

**Affiliations:** 1 College of Animal Science and Technology, Sichuan Agricultural University, Chengdu 611130, China; 2 Key Laboratory of Livestock and Poultry Multi-Omics, Ministry of Agriculture and Rural Affairs, Sichuan Agricultural University, Chengdu 611130, China; 3 Farm Animal Genetic Resources Exploration and Innovation Key Laboratory of Sichuan Province, Sichuan Agricultural University, Chengdu 611130, China; 4 Sichuan Dekang Agriculture and Animal Husbandry Food Group Co., Ltd., Chengdu 610200, China

**Keywords:** spermidine, intestine, metabolite, microorganisms, intestinal morphology

## Abstract

Spermidine is a class of biologically active organic small molecules that play an important role in maintaining intestinal homeostasis. The specific objective of this study was to explore the effects of spermidine on intestinal morphology, metabolites, and microbial diversity in mice. We showed that 0.3 mmol/L of spermidine significantly promoted the growth of ileal villi (*p* < 0.05), and 3.0 mmol/L of spermidine significantly increased the body weight of mice and promoted the growth of jejunum villi (*p* < 0.05). The 16S rDNA sequencing results indicated that 3.0 mmol/L of spermidine affected the balance of the intestinal flora by increasing the abundance of intestinal Lactic acid bacteria and reducing the abundance of harmful bacteria (*Turicibacter* and *Alistipes*). Additionally, spermidine affects the levels of microbial metabolites such as succinic acid and *Pantetheine*. In summary, spermidine affects intestinal morphology and regulates intestinal flora and metabolites, and this study has provided a new understanding of spermidine’s effects on the intestinal tract.

## 1. Introduction

Spermidine is a positively charged aliphatic small molecule compound that is widely present in living organisms [1]. Spermidine can participate in the regulation of a variety of biological processes, such as the regeneration of tissue, signal transduction, and ion channel regulation [2,3]. The small intestine is the primary organ for digesting food and absorbing nutrients, and its state directly affects the growth rate of the body [4]. Polyamines, as intestinal maturation factors, can regulate intestinal development, metabolism, and other physiological processes in animals [5], and some recent studies have focused on the effects of spermine and putrescine on animal intestinal morphology. Spermine increases jejunal villus height and crypt depth and promotes intestinal development in weaned rats [6]. The addition of putrescine to their diet significantly improved intestinal digestion and absorption ability in piglets [7]. However, the effects of spermidine on intestinal morphology in mice is still unclear. There are many microorganisms in the animal intestine that can maintain the basic function of the gut and affect the healthy development of the body [8]. Some metabolites secreted by gut microbes can also improve gut health. Zhang et al. found that replenishing putrescine can improve the microflora balance in the colon contents and reduce the degree of diarrhea in piglets [9]. Liu et al. found that spermine supplementation can regulate intestinal metabolism in piglets and increase the number of beneficial bacteria, thus enhancing intestinal health [10]. Therefore, spermidine intake can likely affect intestinal flora colonization and metabolic characteristics.

Spermidine has greater effects on some biological processes than putrescine. It is necessary to investigate whether spermidine can also play a larger role than putrescine in promoting intestinal health. Therefore, in this study, different concentrations of spermidine were added to the drinking water of mice, and the effects of spermidine on the intestinal health of the mice were explored by HPLC, 16S rRNA, and metabolite sequencing.

## 2. Materials and Methods

### 2.1. Animals and Experimental Protocols

Six-week-old ICR (Institute of Cancer Research) female mice were purchased from Chengdu Dashuo Experimental Animal Co., Ltd. (Chengdu, China). The spermidine added to their drinking water was purchased from Sigma. The mice were weighed and randomly divided into a control group, an LSPD group (low spermidine) and an HSPD group (high spermidine) (*n* = 12). All ICR mice were placed in plastic cages containing shavings, and the temperature was kept at 22 ± 2 °C, with 55% humidity. The mice were given feed pellets and water ad libitum. Then, 0.3 mmol/L of spermidine was added to the drinking water of the LSPD group, and 3.0 mmol/L of spermidine was added to the drinking water of the HSPD group. After feeding for 90 days, all mice were moved to the Animal Physiology Laboratory, where the mice were killed by cervical dislocations and their duodenums, jejunums, ileums, and colons were quickly colonized. After the intestinal tissues were washed with normal saline, the intestinal tissues of 3 mice were randomly taken and fixed in fixative, and the rest of the tissue samples were placed in a −80 ℃ refrigerator until use. The contents of the colons and cecums were separated and used for polyamine content detection, metabonomic sequencing, and microbial diversity omics detection after being frozen using liquid nitrogen. The care and use of the animals were maintained in accordance with the Animal Welfare Guide, and all test plans were approved by the Animal Care and Use Committee of Sichuan Agricultural University.

### 2.2. Polyamine Content Measurement 

The polyamine contents of the mice intestines were determined according to the method of Kang et al. [11]. Intestinal samples were placed into HClO_4_, 1,6-hexanediamine (Sigma, Shanghai, China) was added, and the samples were ground. Then, the samples were lysed by ultrasound and centrifuged, and the supernatant was collected. HClO_4_ was added and the above steps were repeated. After the supernatant was adjusted to an alkaline pH, 1.4 μL of benzoyl chloride was added, and it was protected from light in a 40 °C water bath for 1 h. The sample was adjusted to a neutral pH. The derivate was separated by a HyperSep C18 (Thermo Fisher Scientific, Waltham, MA, USA). The sample was eluted with methanol (Sigma-Aldrich, Shanghai, China) and filtered into a 1.5 mL EP tube for storage until detection. 

### 2.3. Intestinal Histo-Morphological Detection

The collected intestinal tissue was washed with normal saline, and the filter paper was dried and fixed in 4% paraformaldehyde. The sample was dehydrated with ethanol, cleared with anhydrous ethanol xylene, and embedded in paraffin. The 4 μm-thick sections were fixed on the slide and stained with hematoxylin eosin. The prepared sections were observed under a light microscope, and the villus height and recess depth of each intestinal segment were photographed and recorded.

### 2.4. Detection of Inflammatory Cytokines in the Colon

The DNA of the mouse colon contents was extracted according to the instructions of the TGuide S96 magnetic bead method soil/fecal genomic DNA extraction kit (Tiangen Biochemical Technology Co., Ltd., Beijing, China). PCR amplification, purification, and construction of the library were carried out for the V3-V4 region. The samples were sequenced on an Illumina Novaseq6000 platform. The construction of the relevant library and high-throughput sequencing were completed by Biomarker Technologies Biotechnology Co., Ltd. (Beijing, China).

### 2.5. Metabonomic Analysis of Colonic Contents

The intestinal samples were ground and L-2-chlorophenylalanine was added. Then, the samples were centrifuged at 10,000 rpm for 15 min at 4 °C, and the supernatant was transferred to a sample bottle after standing. Relative quantification of the colon metabolites was performed with a Waters Liquid Chromatograph (ultra-high performance liquid chromatography) system coupled to a Waters Xevo G2-XS QT high-resolution mass spectrometer (Waters Corp., Milford, MA, USA). Acquity UPLC HSS T3 columns (1.8 um 2.1 ∗ 100 mm) were purchased from Waters. Phase A of the liquid chromatography was set as the water phase and phase B was set as the acetonitrile. MassLynx V4.2 was used to collect the high-resolution mass spectrometry data, select ions (through its data acquisition software (Progenesis QI)), and collect the secondary mass spectrometry data. The raw mass spectrum data were converted to mzXML format using the Proteo Wizard software, and the material was identified using the R program package and a self-built database of secondary mass spectra. The method of combining the difference multiple, the *p*-value, and the VIP value of the OPLS-DA model was adopted to screen the differential metabolites. The screening criteria were an FC of >1, a *p*-value of <0.05, and a VIP of >1. Correlation analysis was performed using BMKCloud (www.biocloud.net)

### 2.6. Statistical Analysis

The MEANS process was performed using SAS 8.0 statistical analysis software for the statistical analysis of the data, and the descriptive process was completed using ANOVA and Duncan multiple comparison analysis to determine the statistically significant relationships. GraphPad Prism 6.0 software was used for map plotting. The results are presented as means ± SEMs.

## 3. Results

### 3.1. Effects of Spermidine on the Litter Weight of the Mice

**Figure 1:**
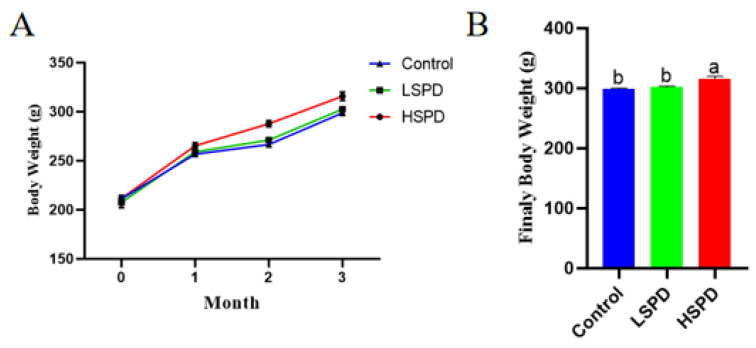
Effect of spermidine on mouse weight gain and final body weight of the mice. (**A**) Evolution of body weight with spermidine. (**B**) Final body weights of the mice consuming spermidine at month 3. a,b: The same row without the same letters indicates a significant difference (*p* < 0.05).

To determine the effect of spermidine on the weight of the mice, the changes in body weight during the first three months and the final body weight were recorded. As shown in Figure 1A, there was no significant difference in initial body weight (in grams) among the treatment groups (control, 211.25 ± 0.9; LSPD, 207.50 ± 5.51; and HSPD, 211.50 ± 3.11; *p* > 0.05). Although the weight of the mice in each group increased gradually, the weight gain was more significant in the HSPD group. As shown in Figure 1B, the body weights of the mice in the HSPD group were significantly higher than those of the control and LSPD groups (*p* < 0.05), but there were no significant differences between the body weights of mice in the LSPD group and those of the control group (*p* > 0.05). The results showed that 3.0 mmol/L spermidine helped the mice gain weight.

### 3.2. Effect of Spermidine on Intestinal Morphology

**Figure 2:**
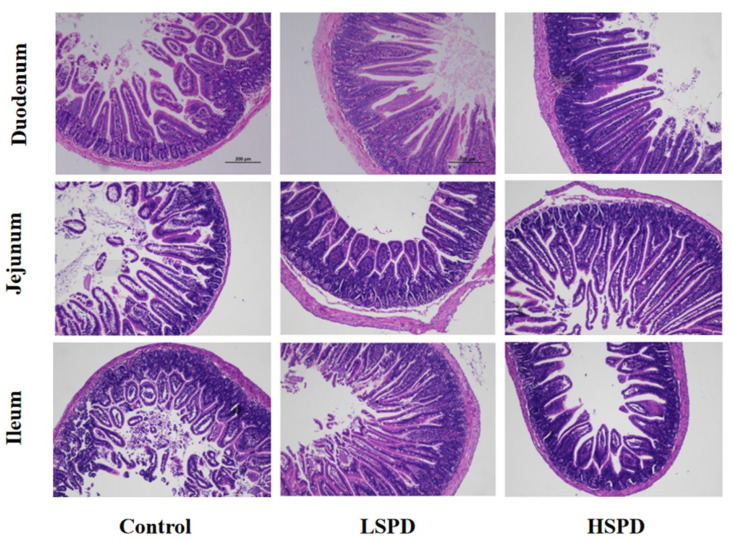
Histological evaluation of small intestine tissues (×100) of the mice after adding spermidine to their drinking water. The scale bar is 200 mm.

To understand the effect of the addition of spermidine on the intestinal morphology of the mice, we observed the changes in the villus height and crypt depth of the mouse intestines under a light microscope (Figure 2). There were no significant differences in villus heights or crypt depths of the duodenums between the spermidine-treated group and the control group (*p* > 0.05), and the villus heights and crypt depths of the jejunums in the HSPD group were significantly higher than those of the control group (*p* < 0.05). In the LSPD group, the villus heights of the ileal tissues were significantly higher than those of the control group (*p* < 0.05) (Table 1). 

### 3.3. Effects of Spermidine on the Intestinal Polyamine Contents of the Mice

**Figure 3:**
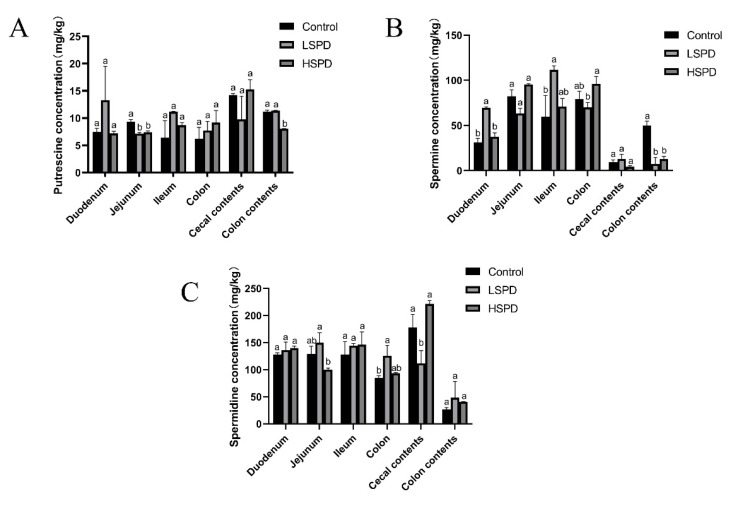
Effects of spermidine on the polyamines in the intestinal tissues of the mice. (**A**) Putrescine. (**B**) Spermine. (**C**) Spermidine. The data are expressed as means ± SEMs. The graph bars marked with different letters represent significant differences between the different groups (*p* < 0.05), whereas the bars labeled with the same letter correspond to results that showed no statistically significant differences.

To study the effect of adding spermidine on the contents of the intestinal polyamines, we detected the polyamine contents of the intestines of the mice using high-performance liquid chromatography (Figure 3A–C). Different doses of spermidine had no significant effect on the putrescine levels of the duodenal, ileal, colonic, or cecal contents (*p* > 0.05). Jejunal putrescine levels were significantly lower in the LSPD group (*p* < 0.05). The putrescine levels of the jejunal and colonic contents in the HSPD group were significantly reduced (*p* < 0.05). Different doses of spermidine had no significant effect on the spermidine levels of the duodenal, jejunum, ileal, or colonic contents (*p* > 0.05). The levels of spermidine in the colons of the LSPD group were significantly increased, and the spermidine levels of the cecal contents were significantly reduced (*p* < 0.05). The intestinal spermidine levels of the LSPD group were significantly changed, while those of the HSPD group were not. The spermine levels in the duodenums and ileums of the LSPD group were significantly increased, and different doses of spermidine significantly reduced the spermidine levels of the colonic contents (*p* < 0.05). 

### 3.4. Effect of Adding SPD to Drinking Water on the Intestinal Microbiota of the Mice

**Figure 4:**
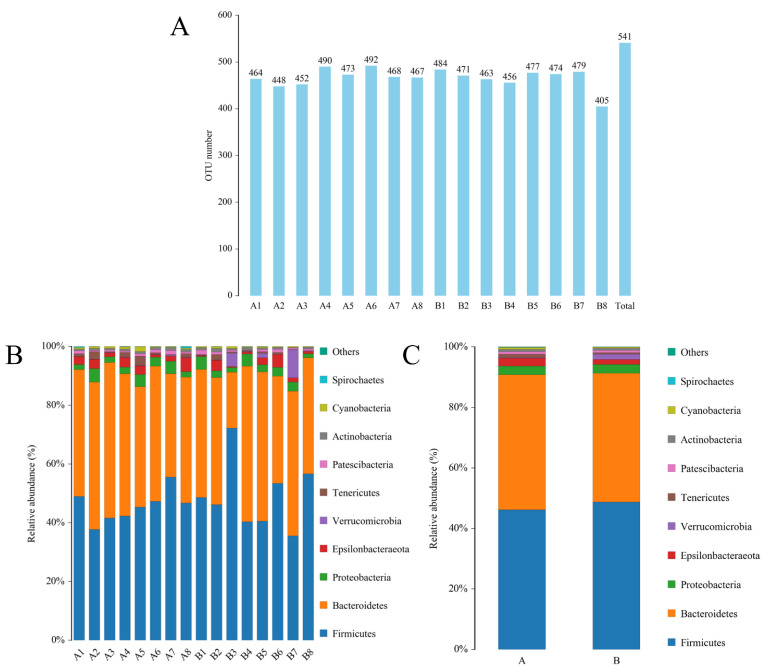

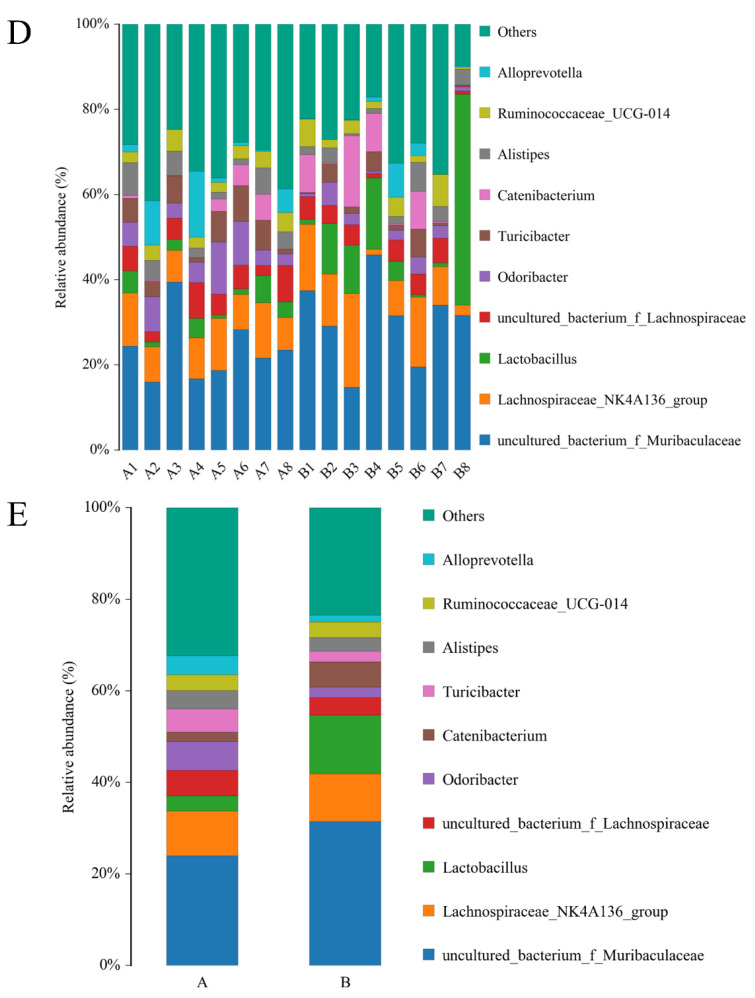

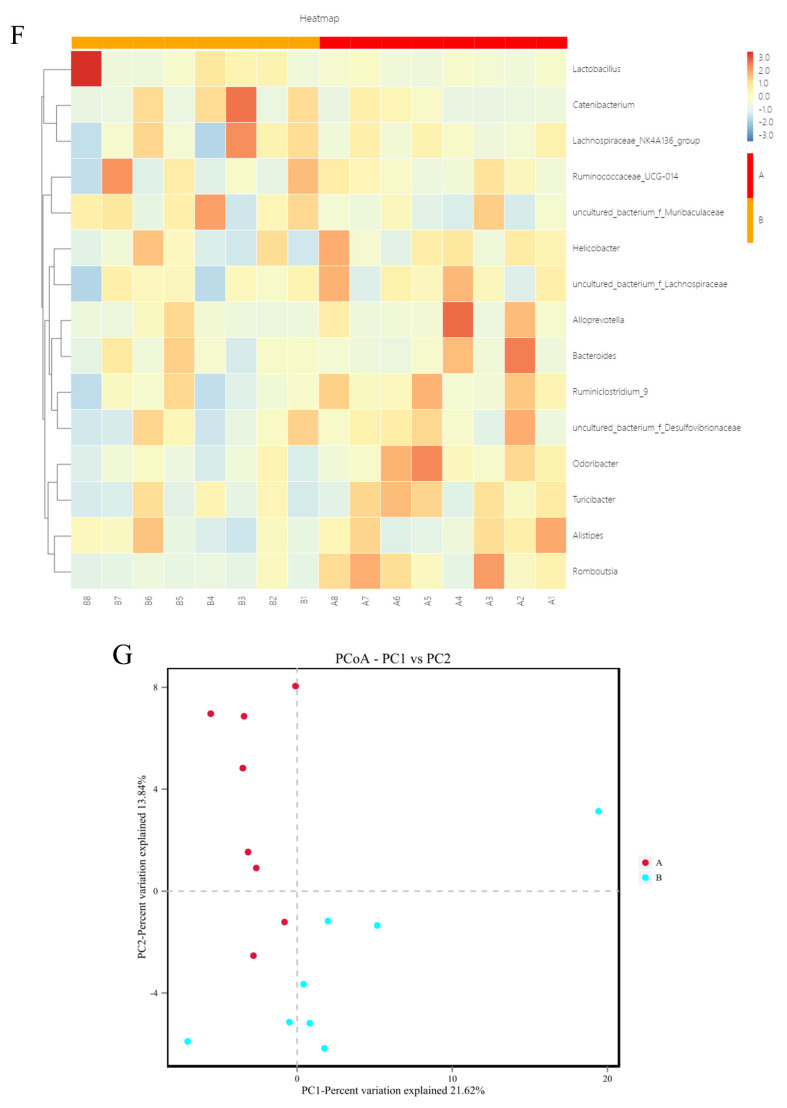
Effects of adding SPD to drinking water on intestinal microbiota in mice. (**A**) OTU quantity statistics. (**B**,**C**) Microbial composition of the mouse intestinal tract at the phylum level. (**D**,**E**) Microbial composition of the mouse intestinal tract at the genus level. (**F**) Heatmap of species clustering at the genus level. (**G**) PCoA of the control group and the 3.0 mmol/L spermidine treatment group.

To further investigate the effects of spermidine on the intestinal tracts of the mice, 16S rRNA sequencing was performed to detect changes in the intestinal microflora of the mice in the control group and the HSPD group. A total of 16 samples were analyzed using an Illumina HiSeq platform, and 1,730,426 tags without primers were generated (101,309 tags per sample, on average). Based on the PCoA, differences in the intestinal microbial community structures were found between the control group and the spermidine treatment group (Figure 4G). The filtered sequences were clustered into 541 OTUs at 97% similarity (Figure 4A). The top ten intestinal microflora were selected to draw a histogram. The microbiota in the intestinal contents of the mice mainly included *Firmicutes*, *Bacteroidetes*, *Spirochetes*, *Cyanobacteria*, *Verrucomicrobia*, *Escherichia coli*, *Actinomycetes*, *Bacteroides*, *Softwall,* and *Proteus*. At the phylum level (Figure 4B,C), in the intestinal contents of the HSPD mice, the levels of *Verrucomicrobia* were higher than those of the control mice, and at the species level, the amounts of *Softwall* and *Escherichia coli* were higher than those of the control mice. At the genus level (Figure 4D,E), compared to the control group, *Muribaculaceaer* and *Lactobacillus* levels were higher and the *Turicibacte*, *Lachnospiraceae,* and *Odoribacter* were lower in the intestinal contents of the HSPD group. Through further analysis of species heatmap clustering, it was found that spermidine also reduced the abundance of bacteria such as *Rombutzia*, *Alistipes,* and *Turicibacter*. This shows that spermidine affects intestinal microbes, and it can effectively regulate intestinal flora and improve the proportion of intestinal flora (Figure 4F). 

### 3.5. Effects of Spermidine on Intestinal Metabonomic Characteristics

**Figure 5:**
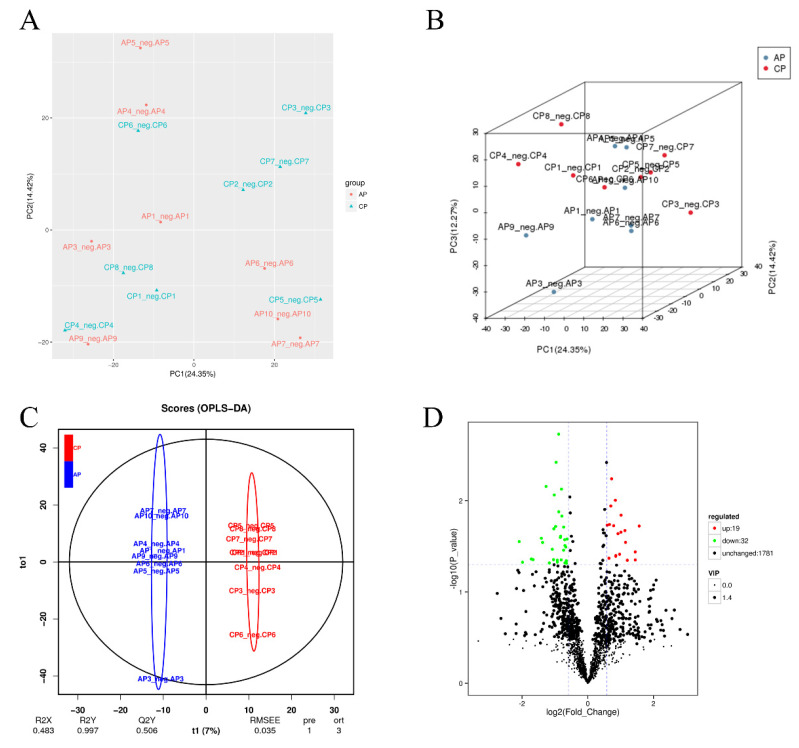

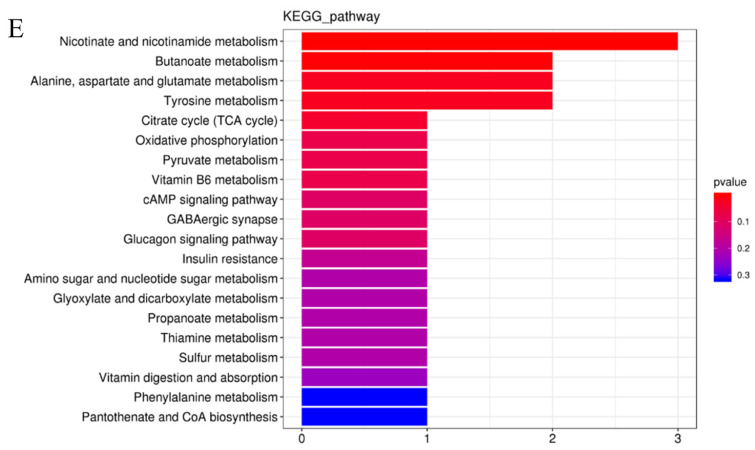
Effects of spermidine on intestinal metabonomic characteristics. (**A**,**B**) PCA of intestinal metabolites in the mice. (**C**) OPLS-DA of intestinal metabolites in the mouse colons. (**D**) Volcano diagram of the differential metabolites in the colon contents of the mice. (**E**) Significantly different metabolites participate in the classification of metabolic pathway enrichment. In this figure, AP is the control group and CP is the HSPD group.

To better understand the effects of spermidine on the gut, we conducted high-throughput sequencing and analyses of the intestinal metabolites in the two groups of mice. Compared with the control group, the spermidine treatment group had better aggregation, indicating that spermidine processing affects gut metabolites (Figure 5A,B). We analyzed the control and HSPD groups by orthogonal partial least square discriminant analysis to further understand the effects of spermidine on intestinal metabolites, and a significant separation between the control and HSPD group was found, which we clustered into two quadrants. It was further suggested that spermidine can regulate the changes in metabolites in the intestinal contents of the mice (Figure 5C). After screening the metabolites in the intestinal contents of the spermidine-treated and control mice, a total of 51 differential metabolites were screened, including 32 upregulated metabolites and 19 downregulated metabolites (Figure 5D, Table 2). Forty-one of the detected differential metabolites belonged to positional differential metabolites without specific corresponding names, and so they are not described in the text. The elevated metabolites after spermidine treatment were succinic acid, maleic acid, pantetheine, etc. The downregulated metabolites were N1-methyl-2-pyridone-5-carboxamide, pyridoxal 5′-phosphate, ribulose 5-phosphate, D-glucosamine 6-phosphate, etc. (Table 3). The KEGG pathway enrichment analysis of the differential metabolites in the feces of the control and HSPD groups showed that the metabolites were primarily concentrated in niacin and nicotinamide, butyrate, alanine, the citric acid cycle, and dicarboxylic acid metabolism, and they mediated the VB6 pathway, cAMP signaling pathway, glucagon pathway, and insulin resistance metabolic pathway (Figure 5E).

## 4. Discussion

Intestinal health is very important to the health of the body, and the body’s digestion and absorption of nutrients depends on the health of the intestines [12]. Polyamines regulate physiological processes such as intestinal development and metabolism. Liu et al. found that adding different concentrations of putrescine to the diet could significantly increase the height of intestinal villi [7]. This is consistent with our study, which showed that after 3 months of adding spermidine to drinking water, the villi heights in the jejunums and ileums of the mice increased significantly. However, duodenal morphology did not change significantly. The duodenum is considered to be the primary site of polyamine absorption [13]. It is presumed that the duodenum has a strong tolerance to polyamine, and therefore, it does not change significantly in morphology. Research has shown that Astragalus polysaccharides may promote the proliferation of intestinal epithelial cells by promoting the production of putrescine [14]. Probiotics can promote weight gain in birds by improving intestinal morphology [15]. Interestingly, the HSPD group showed a significant increase in the heights of the jejunum villi, but the LSPD group showed a significant villi height decrease. The LSPD group showed significant increases in the heights of the ileum villi, while those of the HSPD group did not change significantly. At the same time, we found that spermidine significantly affected gut spermine levels. Xu et al. showed that spermine could promote the proliferation and migration of intestinal epithelial cells [16]. Our study showed that the spermine contents of the jejunums in the LSPD group were significantly lower than those of the control group, while the spermine content of the ileums were significantly higher than those of the control group. The HSPD group did not differ significantly from the control group in the amounts of spermine in the jejunums and ileums. In summary, the effects of spermidine on intestinal morphology are tissue-specific and may be related to changes in the spermine contents of the intestinal tract. Spermine can affect cell differentiation [17]. The spermidine contents of the HSPD group were significantly lower than those of the other two groups. The crypts of the intestines contain stem cells that can differentiate, and the increased crypt depths of the jejuni in the LSPD group may also have been related to the reduced spermine contents. Our study showed that adding spermidine to drinking water significantly increased the body weights of the mice, presumably because spermidine affects spermine levels, which in turn affect the growth of intestinal villi and thus the digestion and absorption of nutrients in the gut. We found that the small intestines had the highest spermidine contents and the lowest putrescine contents. The amounts of spermine in the intestinal contents were lower than those of the small intestines, while the amounts of putrescine in the intestinal contents were the same as those of the small intestines. Eisenberg et al. found that blood and liver levels in older mice increased significantly after providing drinking water containing 3 mmol/L of spermidine for 6 months [18]. Other studies have shown no significant changes in gut polyamines in mice fed physiological doses of spermidine for 26 weeks. Our study showed that spermidine has a very limited effect on gut putrescine and spermidine levels. It was hypothesized that spermidine enters the blood circulation or metabolism from the intestinal tract because of the body’s regulation, and the influence of the intestinal morphology and body weights of the mice may be related to spermidine metabolism. Because we neglected to measure the polyamine levels in the blood of the mice, it remains to be seen where spermidine goes once it enters the body. In summary, the polyamine contents of different intestinal segments were tissue-specific. Spermidine (3 mmol/L) slightly improved intestinal morphology and development, and it promoted weight gain in mice.

Polyamines can promote the proliferation of certain flora and affect the vitality of other intestinal flora [19]. A great variety of microorganisms exist in the guts of animals, and they are involved in regulating the basic functions of the gut and affect the health of the body [20]. We found that the composition and diversity of mouse intestinal microbes were affected by spermidine. *Alistipes* and *Turicibacter* are believed to be pathogenic bacteria associated with colitis. Moschen et al. showed that an increased abundance of *Alistipes* in the gut led to inflammation and tumors [21]. Transplanting the microflora of systemic lupus erythematosus mice into the guts of germ-free mice increased the abundance of *Turicibacter* and the expression of inflammatory factors [22]. Our research showed that spermidine significantly reduced the abundance of *Alistipes* and *Turicibacter* in the guts of the mice, suggesting that the spermidine supplementation had anti-inflammatory effects. Lactic acid bacteria can promote the synthesis of intestinal epithelial cells and secrete immunoglobulin to maintain bacterial homeostasis in the digestive tract [23,24]. Our study showed that spermidine significantly increased *Lactobacillus* content, and it was speculated that spermidine may promote intestinal absorption and immunity. Interestingly, spermidine also reduces the abundance of certain beneficial bacteria. A decrease in the abundance of *Odoribacter* and *Romboutsia* may cause intestinal diseases [25,26]. Combined with the intestinal morphology and body weights of the mice, it was speculated that spermidine had a more promoting effect than an inhibiting effect on beneficial bacteria. In conclusion, spermidine can affect the intestinal tract of the body by changing the contents of various intestinal flora. The beneficial flora can be increased and the harmful flora can be reduced to improve intestinal digestion and absorption ability.

The intestinal flora itself affects intestinal health, and its metabolites are also involved in regulating a variety of physiological processes. We found that the intestinal metabolites of the mice were altered by spermidine, which affected the metabolism of glyoxylic and dicarboxylic acids and tyrosine, the citric acid cycle, vitamin digestion and absorption, and other metabolic pathways. Succinate is an intermediate product of the tricarboxylic acid cycle and has anti-inflammatory effects. Guillon et al. found that succinate could reduce the upregulation of inflammatory factors and metabolic disorders of various physiological processes caused by the influenza A virus [27]. Maleic acid can release H^+^ to promote the hydrolysis of hemicellulose [28]. In this study, spermidine promoted the upregulation of succinate and maleic acid, and it was speculated that spermidine has anti-inflammatory effects and promotes digestion. The colon is metabolized to produce deoxycholic acid, and high concentrations of deoxycholic acid can damage colon epithelial cells, causing colon cancer [29]. Our study showed that deoxycholic acid levels were reduced by spermidine, which suggests that spermidine may protect the intestinal mucosa from damage. The body’s metabolic are affected by alterations in the metabolites of the gut microbiota. Ma et al. found that nicotinamide can inhibit the NF-κB signaling pathway by activating SIRT, thus producing anti-inflammatory effects [30]. Increased levels of succinate and maleic acid can activate the metabolism of niacin and nicotinamide. It is speculated that spermidine can promote the production of nicotinamide by promoting the production of succinic acid and maleic acid, and thus, it can play an anti-inflammatory role. Spermidine can also induce the production of pantetheine and activate the biosynthesis of pantothenate and CoA. Pantothenic acid and CoA are important cofactors in cells that regulate a variety of metabolic pathways. They are also considered to have the potential to be developed as antibiotics. [31,32]. It was suggested that spermidine also has potential as an antibacterial drug. D-Glucosamine 6-phosphate is involved in the insulin resistance pathway, and cardiovascular disease and type 2 diabetes are also affected by it. Our research shows that spermidine inhibited D-Glucosamine 6-phosphate, thus inhibiting the insulin resistance pathway. It was suggested that spermidine has the potential to improve the body’s glucose uptake and utilization. In conclusion, spermidine can regulate intestinal metabolic processes by affecting the metabolites of intestinal flora

## 5. Conclusions

Spermidine increased the height of intestinal villi and promoted weight gain in mice, and 3.0 mmol/L of spermidine had a significant role in promoting intestinal health. Spermidine (3.0 mmol/L) increased the abundance of *Lactobacillus* in the intestine and reduced the abundance of harmful bacteria, such as *Alistites*, *Turicibacter,* and rumen bacteria. Spermidine (3.0 mmol/L) also regulated the production of metabolites such as succinate, maleic acid, and pantetheine and it altered the metabolism of niacin and nicotinamide, pantothenic acid, and CoA; thus, it improved intestinal metabolic capacity.

## Figures and Tables

**Table 1:** Intestinal villus heights, crypt depths, and chorionic gland ratios of the mice treated with spermidine.

Items	Control	LSPD	HSPD
Duodenum			
Villus height, μm	0.31 ± 0.07	0.38 ± 0.01	0.35 ± 0.07
Crypt depth, μm	0.08 ± 0.01	0.1 ± 0.03	0.10 ± 0.01
Villus height: crypt depth	3.88 ± 1.09	3.40 ± 0.99	3.45 ± 0.42
Jejunum			
Villus height, μm	0.34 ± 0.03 ^b^	0.28 ± 0.06 ^c^	0.42 ± 0.01 ^a^
Crypt depth, μm	0.072 ± 0.02 ^b^	0.10 ± 0.01 ^a^	0.10 ± 0.01 ^a^
Villus height: crypt depth	4.56 ± 0.84 ^a^	3.07 ± 0.80 ^b^	3.98 ± 0.37 ^a^
Ileum			
Villus height, μm	0.22 ± 0.02 ^b^	0.30 ± 0.07 ^a^	0.21 ± 0.02 ^b^
Crypt depth, μm	0.08 ± 0.01	0.09 ± 0.01	0.08 ± 0.01
Villus height: crypt depth	2.28 ± 0.42	3.55 ± 0.79	3.00 ± 0.69

^a–c^: The same row without the same letters indicates a significant difference (*p* < 0.05).

**Table 2:** Number of differential metabolites in the colon contents of the spermidine-treated and control groups.

Group Name	All Difference	Down-Regulated	Up-Regulated
Control vs. spermidine-treated	51	32	19

**Table 3:** Partially different metabolites of the colon contents in the spermidine-treated and control groups.

ID	Metabolite Names	*p*-Value	VIP	Regulated
321	Succinate	0.022117	0.323393	Up
504	Maleic acid	0.018546	2.256601	Up
4350	Pantetheine	0.018791	2.261522	Up
1120	N1-Methyl-2-pyridone-5-carboxamide	0.044214	2.036004	Down
3192	Pyridoxal 5′-phosphate	0.047058	1.961114	Down
3351	Ribulose 5-phosphate	0.006978	2.50337	Down
4439	D-glucosamine 6-phosphate	0.025789	2.29682	Down
7282	6k-PGFlalpha-d4	0.034865	2.04618	Down
7761	Deoxycholic-acid	0.030936	2.062915	Down
10518	Adynerin	0.007462	2.55801	Down

## Data Availability

The data is contained within the Supplementary Materials.

## Supplementary Materials

The following supporting information can be downloaded at: https://www.mdpi.com/article/10.3390/nu15030744/s1, Table S1: Body weight data; Table S2: Intestinal morphology data; Table S3: Polyamines content data.

## References

[1] Madeo F., Bauer M.A., Carmona-Gutierrez D., Kroemer G. (2019). Spermidine: A physiological autophagy inducer acting as an anti-aging vitamin in humans?. Autophagy.

[2] Pegg A.E. (2016). Functions of Polyamines in Mammals. J. Biol.Chem..

[3] Alsaleh G., Panse I., Swadling L., Zhang H., Richter F.C., Meyer A., Lord J., Barnes E., Klenerman P., Green C. (2020). Autophagy in T cells from aged donors is maintained by spermidine and correlates with function and vaccine responses. Elife.

[4] Yamauchi K., Tarachai P. (2000). Changes in intestinal villi, cell area and intracellular autophagic vacuoles related to intestinal function in chickens. Br. Poult. Sci..

[5] Fang T., Liu G., Jia G., Zhao H., Chen X., Wu C., Cai J. (2016). Polyamines: Regulation on Intestinal Homeostasis and Possiblie Mechanisms. Chin. J. Animal Nutrition.

[6] Fang T., Jia G., Zhao H., Chen X., Tang J., Wang J., Liu G. (2016). Effects of spermine supplementation on the morphology, digestive enzyme activities, and antioxidant capacity of intestine in weaning rats. Anim. Nutr..

[7] Liu G., Zheng J., Wu X., Xu X., Jia G., Zhao H., Chen X., Wu C., Tian G., Wang J. (2019). Putrescine enhances intestinal immune function and regulates intestinal bacteria in weaning piglets. Food Funct..

[8] Zhou B., Yuan Y., Zhang S., Guo C., Li X., Li G., Xiong W., Zeng Z. (2020). Intestinal Flora and Disease Mutually Shape the Regional Immune System in the Intestinal Tract. Front. Immunol..

[9] Zhang Z., Dong G., Zhang W., Han H. (2011). Nutritional and physiological effects of dietary putrescine supplementation in weaned piglets. Chin. J. Anim. Sci..

[10] Liu G., Mo W., Cao W., Wu X., Jia G., Zhao H., Chen X., Wu C., Wang J. (2020). Effects of spermine on ileal physical barrier, antioxidant capacity, metabolic profile and large intestinal bacteria in piglets. RSC Adv..

[11] Kang B., Jiang D., He H., Ma R., Chen Z., Yi Z. (2017). Effect of Oaz1 overexpression on goose ovarian granulosa cells. Amino Acids.

[12] Ducatelle R., Goossens E., De Meyer F., Eeckhaut V., Antonissen G., Haesebrouck F., Van Immerseel F. (2018). Biomarkers for monitoring intestinal health in poultry: Present status and future perspectives. Vet. Res..

[13] Madeo F., Hofer S.J., Pendl T., Bauer M.A., Eisenberg T., Carmona-Gutierrez D., Kroemer G. (2020). Nutritional Aspects of Spermidine. Annu. Rev. Nutr..

[14] Zhang C.L., Ren H.J., Liu M.M., Li X.G., Sun D.L., Li N., Ming L. (2014). Modulation of intestinal epithelial cell proliferation, migration, and differentiation in vitro by Astragalus polysaccharides. PLoS ONE.

[15] Sefcova M.A., Larrea-Alvarez M., Larrea-Alvarez C.M., Karaffova V., Ortega-Paredes D., Vinueza-Burgos C., Sevcikova Z., Levkut M., Herich R., Revajova V. (2021). The Probiotic Lactobacillus fermentum Biocenol CCM 7514 Moderates Campylobacter jejuni-Induced Body Weight Impairment by Improving Gut Morphometry and Regulating Cecal Cytokine Abundance in Broiler Chickens. Animals.

[16] Xu X., Liu G., Jia G., Zhao H., Chen X., Wu C., Wang J. (2021). Effects of spermine on the proliferation and migration of porcine intestinal epithelial cells. Anim. Biotechnol..

[17] Pegg A.E. (2014). The function of spermine. IUBMB Life.

[18] Eisenberg T., Abdellatif M., Schroeder S., Primessnig U., Stekovic S., Pendl T., Harger A., Schipke J., Zimmermann A., Schmidt A. (2016). Cardioprotection and lifespan extension by the natural polyamine spermidine. Nat. Med..

[19] Ferioli M.E., Pirona L., Pinotti O. (2000). Prolactin and polyamine catabolism: Specific effect on polyamine oxidase activity in rat thymus. Mol. Cell Endocrinol..

[20] Wang X., Xia X., Gong M., Yin Y. (2014). Exogenous Polyamine: Effects on Intestinal Structure and Function in Piglet and Its Mechanism. Chin. J. Anim. Nutr..

[21] Moschen A.R., Gerner R.R., Wang J., Klepsch V., Adolph T.E., Reider S.J., Hackl H., Pfister A., Schilling J., Moser P.L. (2016). Lipocalin 2 Protects from Inflammation and Tumorigenesis Associated with Gut Microbiota Alterations. Cell Host Microbe.

[22] Ma Y., Guo R., Sun Y., Li X., He L., Li Z., Silverman G.J., Chen G., Gao F., Yuan J. (2021). Lupus gut microbiota transplants cause autoimmunity and inflammation. Clin. Immunol..

[23] Wu X. (2018). Effects of Putrescine Supplementation on Intestinal Development, Antioxidant Function, Immunity, and Microbes in Weaned Piglets. Master’s Thesis.

[24] Jung I.L., Kim I.G. (2003). Polyamines and glutamate decarboxylase-based acid resistance in Escherichia coli. J. Biol. Chem..

[25] Mangifesta M., Mancabelli L., Milani C., Gaiani F., de’Angelis N., de’Angelis G.L., van Sinderen D., Ventura M., Turroni F. (2018). Mucosal microbiota of intestinal polyps reveals putative biomarkers of colorectal cancer. Sci. Rep..

[26] Zhang W., Zou G., Li B., Du X., Sun Z., Sun Y., Jiang X. (2020). Fecal Microbiota Transplantation (FMT) Alleviates Experimental Colitis in Mice by Gut Microbiota Regulation. J. Microbiol. Biotechnol..

[27] Guillon A., Brea-Diakite D., Cezard A., Wacquiez A., Baranek T., Bourgeais J., Picou F., Vasseur V., Meyer L., Chevalier C. (2022). Host succinate inhibits influenza virus infection through succinylation and nuclear retention of the viral nucleoprotein. EMBO J..

[28] Jiang K., Fu X., Huang R., Fan X., Ji L., Cai D., Liu X., Fu Y., Sun A., Feng C. (2022). Production of Prebiotic Xylooligosaccharides via Dilute Maleic Acid-Mediated Xylan Hydrolysis Using an RSM-Model-Based Optimization Strategy. Front. Nutr..

[29] Kasbo J., Saleem M., Perwaiz S., Mignault D., Lamireau T., Tuchweber B., Yousef I. (2002). Biliary, fecal and plasma deoxycholic acid in rabbit, hamster, guinea pig, and rat: Comparative study and implication in colon cancer. Biol. Pharm. Bull..

[30] Ma Y., Bao Y., Wang S., Li T., Chang X., Yang G., Meng X. (2016). Anti-Inflammation Effects and Potential Mechanism of Saikosaponins by Regulating Nicotinate and Nicotinamide Metabolism and Arachidonic Acid Metabolism. Inflammation.

[31] Pietrocola F., Galluzzi L., Bravo-San Pedro J.M., Madeo F., Kroemer G. (2015). Acetyl coenzyme A: A central metabolite and second messenger. Cell Metab..

[32] de Vries L.E., Lunghi M., Krishnan A., Kooij T.W.A., Soldati-Favre D. (2021). Pantothenate and CoA biosynthesis in Apicomplexa and their promise as antiparasitic drug targets. PLoS Pathog..

